# Supplementary material for: In vitro reconstitution of minimal human centrosomes
Source: bioRxiv. 2025 Feb 20:2025.02.20.639226. Preprint. [Version 1] doi: 10.1101/2025.02.20.639226 (PMC11870475; doi:10.1101/2025.02.20.639226)
Supplement: 1 [file NIHPP2025.02.20.639226V1-supplement-1.pdf]

bioRxiv preprint doi: https://doi.org/10.1101/2025.02.20.639226; this version posted February 23, 2025. The copyright holder for this preprint (which was not certified by peer review) is the author/funder, who has granted bioRxiv a license to display the preprint in perpetuity. It is made available under aCC-BY 4.0 International license.

**FIGURE S1. Assembly of CDK5RAP2 scaffolds using different PEG concentrations and different crowders.**

A) 120nM purified GFP::CDK5RAP2 combined with various PEG concentrations (0%, 3%, 6%, 9%)(w/v). Scale bar, 5µm.

B) Quantification of panel 1B. Significant differences were assessed using One-way ANOVA followed by Tukey's multiple comparisons test. Y-axis represents integrated fluorescence intensity(area x mean intensity) of CDK5RAP2 assemblies generated at various PEG concentrations.

C) Micron scale assemblies of GFP::CDK5RAP2 generated using 10% (w/v) PVP, Dextran, Lyzozyme or Ficoll. Scale bar, 5µm.

D) Quantification of panel 1C (mean +/- 95% C.I.; PVP (n=69 assemblies), Dextran (n=79 assemblies), Lyzozyme (n=173 assemblies), Ficoll (n=105 assemblies), PEG (n=130

bioRxiv preprint doi: https://doi.org/10.1101/2025.02.20.639226; this version posted February 23, 2025. The copyright holder for this preprint (which was not certified by peer review) is the author/funder, who has granted bioRxiv a license to display the preprint in perpetuity. It is made available under aCC-BY 4.0 International license.

assemblies)) Y-axis represents integrated fluorescence intensity (area x mean intensity) of CDK5RAP2 represented in Log10 scale.

**FIGURE S2. CDK5RAP2 phospho-site information**

A) Venn diagram indicates shared and unique p-Sites in CDK5RAP2 reported in the phospho-proteome database PhosphoSite Plus (PSP)(Blue), the phospho-sites found in our study (Pink) and the phospho-sites found in both (Purple).

B) List of annotated phospho-sites in PhosphoSite Plus (Hornbeck et al., 2015). Annotated residues also found in our study are in purple.

bioRxiv preprint doi: https://doi.org/10.1101/2025.02.20.639226; this version posted February 23, 2025. The copyright holder for this preprint (which was not certified by peer review) is the author/funder, who has granted bioRxiv a license to display the preprint in perpetuity. It is made available under aCC-BY 4.0 International license.

**FIGURE S3. Regression analysis of 3C asters and microtubule nucleation capabilities of CDK5RAP2 alone**

A) 167nM GFP::CDK5RAP2(WT) scaffolds assembled using anti-CM2 IgMs in the presence of 13µM Hi-Lyte-labeled α/β tubulin mix (25mM HEPES, 50mM KCl, pH7.4).
B) Linear regression and correlation analysis of CDK5RAP2(WT) assemblies. γ-TuRC partitioning (X-axis) is plotted against α/β tubulin partitioning (Y-axis) within any given CDK5RAP2 assembly. Each data point represents a CDK5RAP2 scaffold (n=95).
C) Linear regression and correlation analysis of CDK5RAP2(F75A) assemblies. γ-TuRC partitioning (X-axis) is plotted against α/β tubulin partitioning (Y-axis) within any given CDK5RAP2 assembly. Each data point represents a CDK5RAP2 scaffold (n=117).

bioRxiv preprint doi: https://doi.org/10.1101/2025.02.20.639226; this version posted February 23, 2025. The copyright holder for this preprint (which was not certified by peer review) is the author/funder, who has granted bioRxiv a license to display the preprint in perpetuity. It is made available under aCC-BY 4.0 International license.

**FIGURE S4. Additional analyses of CDK5RAP2 assemblies containing HSET**

A) Regression analysis of 4C (+HSET) vs 3C (-HSET) asters. γ-TuRC partitioning (X-axis) is plotted against α/β tubulin partitioning (Y-axis). Each data point represents a GFP-CDK5RAP2 scaffold (3C, n=95; 4C, n=133).

B) mCherry-HSET can recruit α/β tubulin to GFP-CDK5RAP2 assemblies in the absence of GTP and glycerol at 23°C. Reactions contain 581nM GFP-CDK5RAP2, 583nM mcherry-HSET, 16μM Hi-Lyte-labeled α/β tubulin mix, anti-CM2 IgM. Buffer conditions are: 25mM HEPES, 50mM KCl, 666nM ATP, 16.65mM $MgCl_2$ pH7.4. Scale bar, 3μm.

C) Quantification of clusters and CDK5RAP2 scaffolds per cluster in ATP+/- conditions. Clusters are defined as local collections of CDK5RAP2 scaffolds connected via microtubule asters. ATP- (8 clusters from 5 images), ATP+ (25 clusters from 5 images).

D) Area of GFP::CDK5RAP2 fluorescence at 4C scaffolds (mean +/- 95% C.I.; ATP, n=36 assemblies; ATP+, n=87 assemblies). Significant differences were assessed using a student t-test.

E) Aspect ratio of 4C scaffolds (mean +/- 95% C.I.; ATP, n=36 assemblies; ATP+, n=87 assemblies). Significant differences were assessed using a student t-test.

(which was not certified by peer review) is the author/funder, who has granted bioRxiv a license to display the preprint in perpetuity. It is m
available under aCC-BY 4.0 International license.

# Figure S1

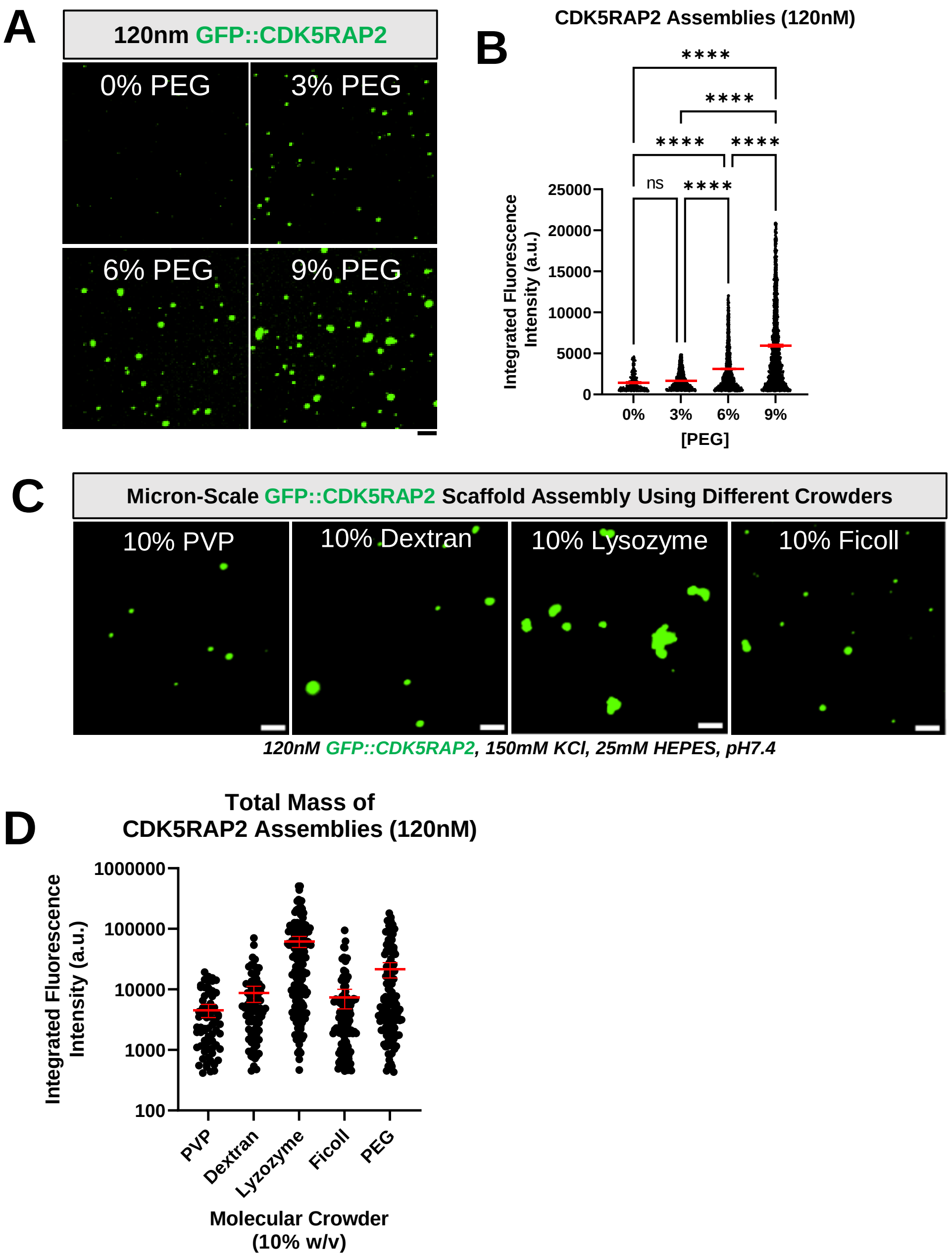

(which was not certified by peer review) is the author/funder, who has granted bioRxiv a license to display the preprint in perpetuity. It is m
available under aCC-BY 4.0 International license.

# Figure S2

A

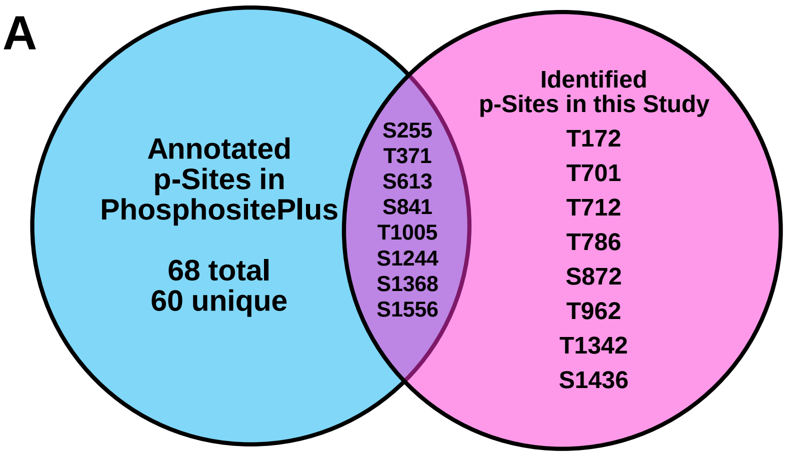


B

| Annotated CDK5RAP2 p-Sites on PhosphoSite Plus | | | | | | |
|---|---|---|---|---|---|---|
| 102 | 371* | 547 | 732 | 1020 | 1238 | 1428 |
| 128 | 374 | 548 | 791 | 1061 | 1244* | 1488 |
| 140 | 391 | 550 | 815 | 1074 | 1343 | 1490 |
| 161 | 392 | 564 | 841* | 1076 | 1349 | 1548 |
| 255* | 400 | 576 | 945 | 1077 | 1350 | 1556* |
| 274 | 461 | 613* | 947 | 1102 | 1354 | 1617 |
| 324 | 463 | 682 | 952 | 1111 | 1360 | 1666 |
| 360 | 466 | 697 | 1001 | 1164 | 1362 | 1885 |
| 366 | 468 | 698 | 1005* | 1172 | 1368* | |
| 369 | 486 | 706 | 1017 | 1173 | 1389 | |

*Annotated p-sites also found in our study

(which was not certified by peer review) is the author/funder, who has granted bioRxiv a license to display the preprint in perpetuity. It is m
available under aCC-BY 4.0 International license.

# Figure S3

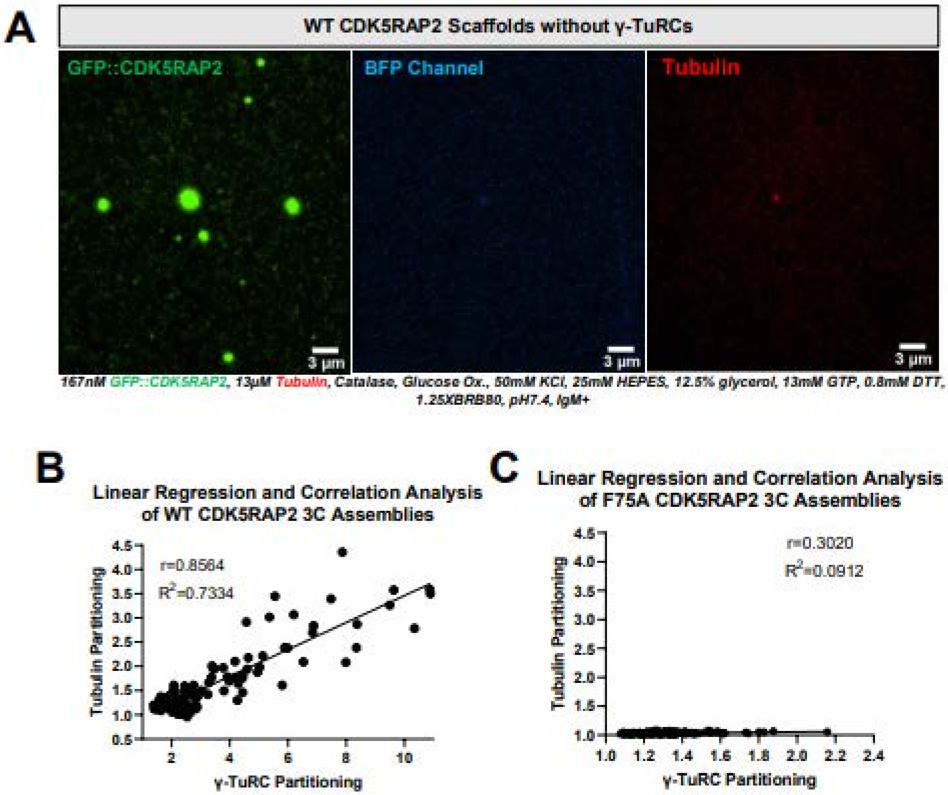

(which was not certified by peer review) is the author/funder, who has granted bioRxiv a license to display the preprint in perpetuity. It is m
available under aCC-BY 4.0 International license.

# Figure S4

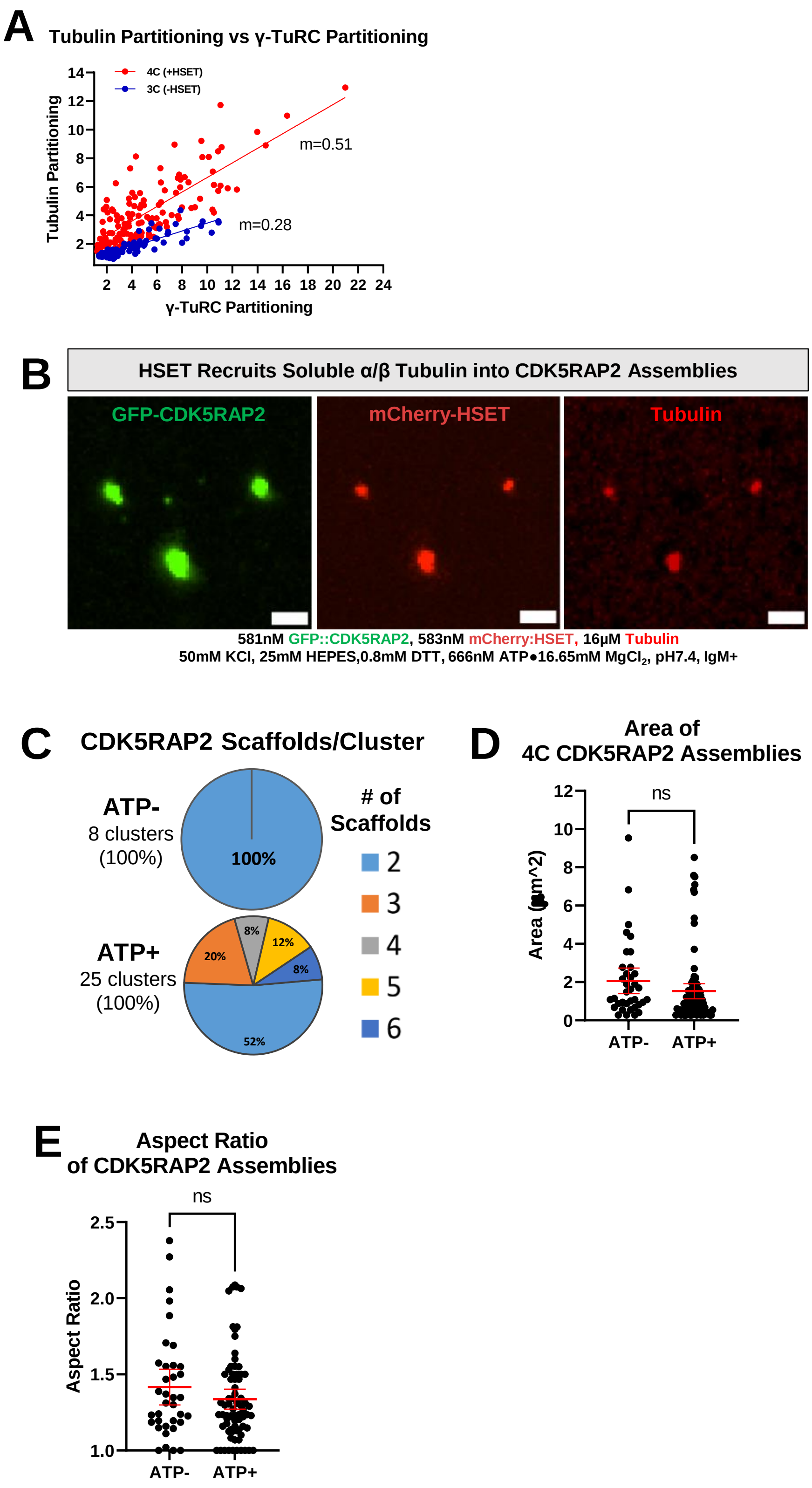

(which was not certified by peer review) is the author/funder, who has granted bioRxiv a license to display the preprint in perpetuity. It is m
available under aCC-BY 4.0 International license.

# Table S1

| Descriptive Statistics of GFP::CDK5RAP2 Assemblies (125nm) Using Anti-CM2 IgM | | | | | |
|---|---|---|---|---|---|
| **N=166** | **Area (µm^2)** | **Mean Int.** | **Max Int.** | **Circularity** | **Diameter (µm)** |
| Minimum | 0.7510 | 3518 | 3924 | 0.7790 | 1.226 |
| Maximum | 7.323 | 31853 | 64601 | 1.000 | 3.494 |
| Range | 6.572 | 28335 | 60677 | 0.2210 | 2.268 |
| Mean | 1.875 | 8698 | 15068 | 0.9887 | 1.835 |
| Std. Deviation | 1.429 | 4397 | 10830 | 0.03598 | 0.5865 |
| Std. Error of Mean | 0.1109 | 341.2 | 840.6 | 0.002793 | 0.04552 |

(which was not certified by peer review) is the author/funder, who has granted bioRxiv a license to display the preprint in perpetuity. It is m
available under aCC-BY 4.0 International license.

# Table S2

| PROTEIN | JWV | pOCC Vector | Purification Tags (PTs) | Fluorescent Tags |
|---|---|---|---|---|
| CEP215/CDK5RAP2 (FL) | 92 | pOCC29 | N-term MBP-PreScission, C-term PreScission-6xHis | N-term eGFP |
| CEP215/CDK5RAP2 (F75A) | 145 | pOCC29 | N-term MBP-PreScission, C-term PreScission-6xHis | N-term eGFP |
| CEP215/CDK5RAP2 ΔCM2 (Δ1715-1814) | 154 | pOCC29 | N-term MBP-PreScission, C-term PreScission-6xHis | N-term eGFP |
| HSET | 151 | pOCC195 | C-term PreScission-6xHis | C-term mCherry |
| HSET(ΔIDR) (Δ2-150) | 152 | pOCC195 | C-term PreScission-6xHis | C-term mCherry |
| PLK-1(T210D) | 142 | pOCC7 | C-term PreScission-6xHis | None |
| KD PLK-1 (K82A) | 144 | pOCC7 | C-term PreScission-6xHis | None |
| | | | | |
| **PROTEIN** | **JWB** | **Plasmid Name/Number** | **Purification Tags (PTs)** | **Fluorescent Tags** |
| mCherry | 68 | pET AviTag His6 mCherry LIC cloning vector, Plasmid #29722 | N-term 6xHis-TEV | Itself |
